# A case report of a spherical mass in the right atrium: myxoma or thrombus?

**DOI:** 10.3389/fonc.2025.1581972

**Published:** 2025-04-16

**Authors:** Yulian Wu, Xidan Wang, Daoling Yang, Xiaoying Tao

**Affiliations:** Department of Ultrasound, JinHua Municipal Central Hospital, Jinhua, Zhejiang, China

**Keywords:** right heart thrombus, inferior vena cava thrombus, myxoma, echocardiography, cardiac magnetic resonance imaging (CMR)

## Abstract

**Background:**

Intracardiac masses encompass a spectrum of pathologies, including tumors, thrombi, and other proliferative lesions, with left atrial involvement being more common than right atrial involvement. In particular, spherical thrombi in the right atrium are exceedingly rare. Diagnostic evaluation relies on modalities such as transesophageal echocardiography (TEE), cardiac magnetic resonance imaging (CMR), and multidetector computed tomography (MDCT). TEE provides detailed information regarding the mass’s location, number, size, and mobility, while CMR and MDCT offer insights into tissue characterization. In this report, we describe a case in which both TEE and CMR misdiagnosed a spherical thrombus as a myxoma. By analyzing the features of TEE and CMR, we summarize the reasons for this misdiagnosis, aiming to serve as a cautionary reminder for clinicians.

**Case summary:**

We report a case of a 59-year-old male, whose past medical history was notable only for a childhood lower extremity trauma (details unknown and not requiring hospitalization or treatment) and no history of diabetes, hypertension, prolonged immobilization, or familial diseases. A spherical mass was incidentally detected in the right atrium during a routine examination. Initial transesophageal echocardiography (TEE), including three-dimensional imaging, revealed a hyperechoic mass with a distinct stalk attached to the interatrial septum near the inferior vena cava, findings that were initially interpreted as consistent with a myxoma. However, subsequent surgical resection and histopathological analysis demonstrated fibrous tissue proliferation and collagenization, confirming the lesion as a thrombus. The unique spherical configuration and its location underscore the potential for misdiagnosis when relying solely on conventional imaging modalities.

**Conclusion:**

Right atrial thrombi are rare findings observed on echocardiography. This case illustrates an incidental spherical thrombus located near the inferior vena cava entrance at the top of the right atrium. The echocardiographic features of this thrombus can resemble those of a myxoma, necessitating careful differentiation through additional examinations. In this case, the misdiagnosis on TEE was attributed to the mass displaying slightly increased echogenicity, a narrow attachment to the right atrium near the inferior vena cava, and a degree of mobility. Typically, thrombi appear hypoechoic; however, the slightly elevated echogenicity observed here may be due to the chronicity of thrombus formation, which could also account for the narrow attachment. According to the PLACE-T scoring system, the following points were assigned:

- P (Patient history): 0 points.
- L (Lobulation): Lobulated contour, 0 points.
- Attachment site width: Narrow stalk (base diameter/maximal diameter <0.3), +2 points.
- Clinical context: No relevant medical history, 0 points.
- Echogenicity pattern: Heterogeneous echogenicity, +1 point.
- T (Tissue characterization): No specific features, 0 points.
- With a total score of 3 points, the probability of a thrombus is high (sensitivity 92% and specificity 85% for scores ≤3). When TEE is not feasible or yields uncertain findings, other non-invasive imaging modalities such as multi-slice spiral CT (MDCT) or cardiac magnetic resonance imaging (CMR) may be considered. Although these techniques are predominantly used for left atrial assessment—MDCT, for instance, can successfully identify left atrial thrombus with a negative predictive value of 100% and a positive predictive value ranging from 41% to 92%—the accuracy of differentiating right atrial masses remains uncertain. Therefore, in similar cases, it is imperative to integrate the patient’s clinical history, multiple auxiliary examination results, and the PLACE-T score rather than relying solely on the features observed on TEE.

## Highlights

- Spherical chronic thrombus in the right atrium must be differentiated from a myxoma.
- When the imaging features of spherical thrombi and myxomas are partially similar and potentially confusing, the PLACE-T score should be incorporated for comprehensive assessment.
- Accurate identification of rare right atrial thrombi is crucial for determining the appropriate treatment plan.

## 1 Introduction

Intracardiac thrombi primarily occur in the left heart system, which right atrial thrombi are less common. There are two potential sources of right heart thrombi (1): migratory thrombi, also known as active thrombi, which originatie from the superior or inferior vena cava or peripheral deep veins and become lodged within right heart structures en route to the lungs (2); autologous thrombi, also referred to as inactive thrombi, which form within the right heart due to blood flow stasis, particularly in the context of right heart failure. This report describes a case of a right atrial thrombus, the specific type of which remains undetermined. Echocardiography revealed a slightly hyperechoic, roundish mass at the entrance of the inferior vena cava into the right atrium, suggestive of a myxoma, however, pathology confirmed it as a thrombus. Although intracardiac thrombi are not uncommon, the presentation of a spherical thrombus in the right atrium is exceedingly rare. Its unique, round morphology can closely mimic that of a right atrial myxoma, leading to significant diagnostic challenges. Conventional imaging modalities, such as transesophageal echocardiography and cardiac MRI, may reveal overlapping features that complicate the differential diagnosis. Therefore, a heightened clinical suspicion, along with a multimodality imaging approach, is essential for accurate identification. This case not only contributes to the sparse literature on right atrial spherical thrombosis but also underscores the critical need to carefully evaluate intracardiac masses with ambiguous characteristics.

## 2 Case presentation

A 59-year-old male with no significant past medical or other disease history presented to our cardiothoracic surgery department following the discovery of a cardiac mass during a routine ultrasound at an external hospital 23 days prior. The patient reported no discomfort and had not received any specific treatment; his vital signs, as well as cardiopulmonary and abdominal examinations, were within normal limits. The right atrial mass was incidentally identified during this evaluation. His medical history was largely unremarkable, with the exception of a vague childhood history of lower limb trauma that did not necessitate hospitalization, and he had no family history of cardiovascular or thrombotic disorders, diabetes, hypertension, or prolonged immobilization. Detailed laboratory evaluations further supported the diagnosis and differential diagnosis of the cardiac mass: prothrombin time (PT) was 10.8 s (normal control PT: 11.5 s), INR 0.94, prothrombin activity 111.00%, fibrinogen 3.53 g/L, activated partial thromboplastin time (APTT) 31.4 s, thrombin time 15.4 s, and D-dimer level 169 μg/L; additionally, the eosinophil count was 0.53×10^9^/L.

Laboratory tests indicated elevated uric acid (435.0 μmol/L, normal range 208-428 μmol/L), elevated triglycerides (2.64 mmol/L, normal <1.70 mmol/L), and elevated apolipoprotein B (1.15 g/L, normal range 0.60-1.10 g/L). Hepatitis B surface antibody was positive, eosinophil percentage was 8.5%, the eosinophil count was 0.53×10^9^/L and plateletcrit was 0.300%. Other laboratory values are presented in **Table 1**.

**Table 1:** Laboratory values of interest at the time of presentation.

Laboratory values	Reference range	Level at admission
White Blood Cells	3.5-9.5 ×10^9/L	8.78 ×10^9/L
C-reactive Protein	<0.50 mg/L	<8.0 mg/L
Red Blood Cells	4.30-5.80 ×10^12/L	4.45 ×10^12/L
Hemoglobin	130-175 g/L	144 g/L
Creatinine	40.0-135 umol/L	83.0 umol/L
Urea Nitrogen	3.20-7.14 mmol/L	5.45 mmol/L
ALAT	9.0-50.0 U/L	26.3 U/L
ASAT	15.0-40.0 U/L	27.3 U/L
D-dimer	<500 ug/L	169 ug/L
INR	0.85-1.15	0.94
Total Bilirubin	2.0-25.0 umol/L	13.5 umol/L
Direct Bilirubin	<10.0 umol/L	1.9 umol/L
Indirect Bilirubin	–	11.6 umol/L
Alpha-fetoprotein	0-9 ng/mL	3.60 ng/mL
Carcinoembryonic Antigen	0-5 ng/mL	1.13 ng/mL

Timeline		
Time	Location	Events
2023-11-18	Cardiothoracic Surgery Outpatient	Routine checkup detected a cardiac mass. TTE and 3D TEE suggested a heterogeneous mass near the right atrial top.
2023-11-23	Radiology	MRI suggested a benign nodule at the top of the right atrium near the IVC, possibly a myxoma.
2023-11-27	Cardiothoracic Surgery Inpatient	Vital signs normal, normal cardiopulmonary auscultation. Duplex ultrasound showed normal flow in bilateral lower limb and jugular veins. Brain MRI and DWI were normal.
2023-11-28	Radiology	Chest CT revealed multiple small nodules in both lungs.
2023-11-29	Radiology	Coronary CT showed mild stenosis of the LAD, myocardial bridge. Brain MRA was normal.
2023-12-01	Operating Room	Open-heart surgery revealed a black, spherical mass near the IVC opening, size 1cm x 0.8cm. Pathology confirmed a thrombus.
2023-12-07	ICU	Transferred to ICU postoperatively.
2023-12-14	Discharge	Discharged in good condition. Follow-up was normal.

**Figure 1:**
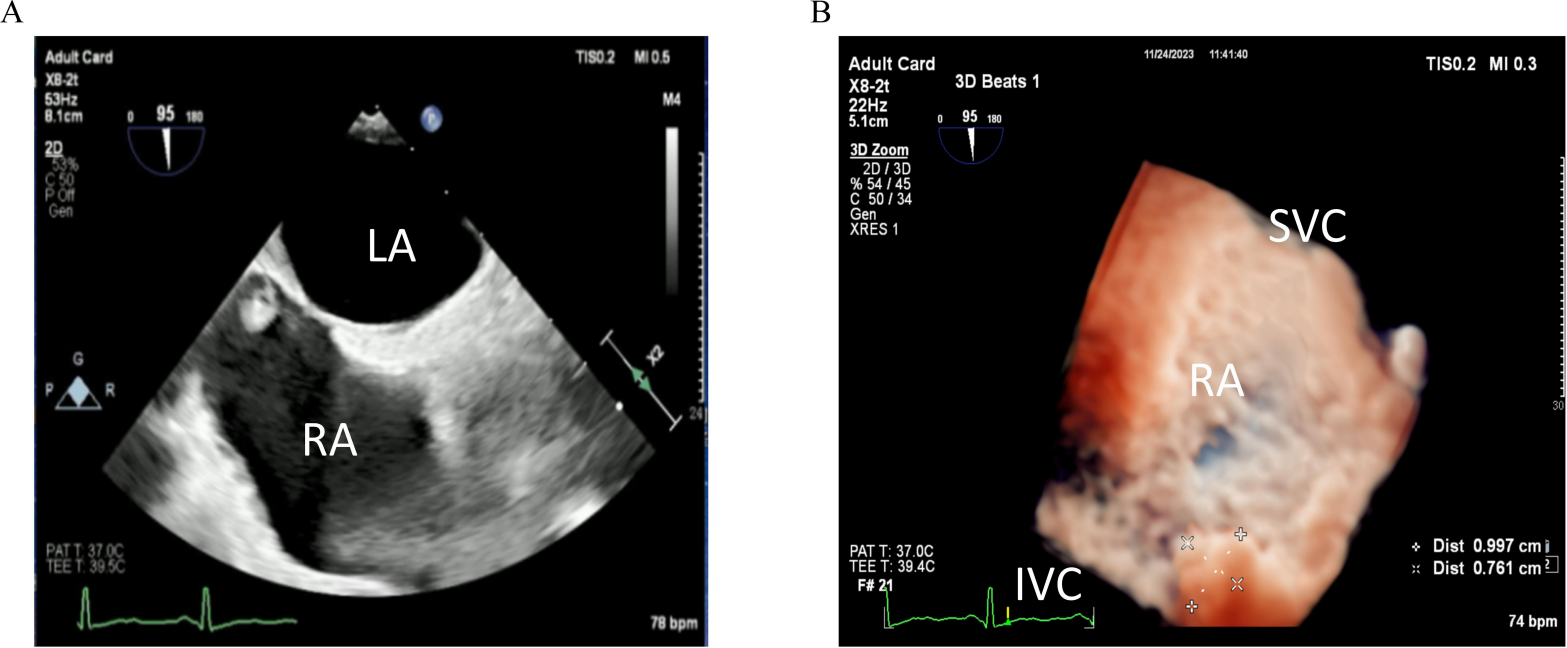
**(A)** Two-dimensional transesophageal echocardiography image obtained before admission shows a slightly echogenic nodule near the entrance of the inferior vena cava at the top of the right atrium (arrow). **(B)** Three-dimensional transesophageal echocardiography image obtained before admission shows a nodule near the entrance of the inferior vena cava at the top of the right atrium (arrow).

Transthoracic echocardiography revealed that the sizes of the cardiac chambers were within normal limits and left ventricular function was normal,with a left ventricular ejection fraction of 67%. A small amount of mitral regurgitation was noted. A slightly hyperechoic mass approximately 10×9 mm in size was observed near the inferior vena cava at the top of the right atrium, suggesting transesophageal echocardiography (TEE) for further evaluation. Three-dimensional TEE revealed a slightly hyperechoic mass approximately 10×8 mm located on the interatrial septum near the entrance of the inferior vena cava in the right atrium, appearing to be attached to the septum by a stalk (**Figures 1A, B**; **Supplementary Video 1**), which raise the possibility of a myxoma.

**Figure 2:**
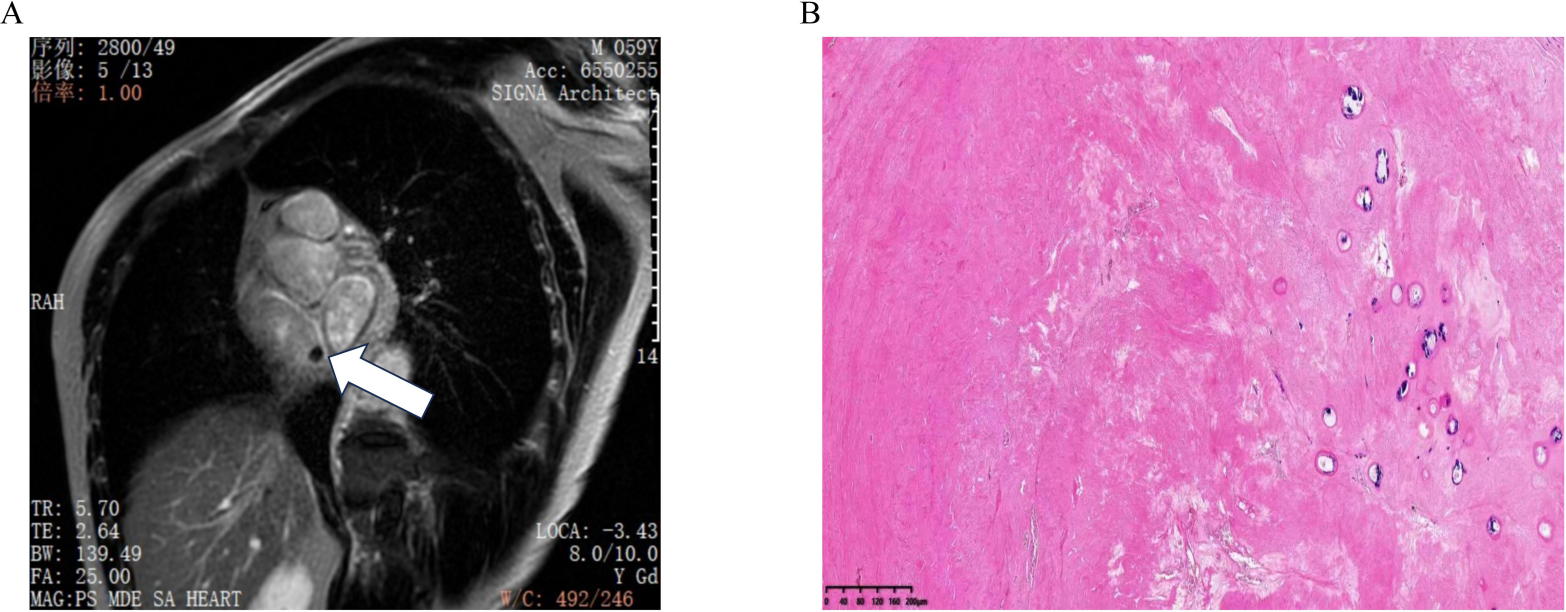
**(A)** Cardiac MRI shows a nodule at the top of the right atrium near the opening of the inferior vena cava, with no enhancement observed after contrast administration. **(B)** Pathology result shows fibrous tissue proliferation and collagenization, consistent with thrombus tissue.

Cardiac MRI was performed to further characterize the mass in the right atrium. MRI demonstrated a nodule with a short T2 signal approximately 8 mm in size, located near the inferior vena cava opening at the top of the right atrium. The nodule echibited clear boundaries and no significant enhancement following contrast administration, suggesting a benign lesion with the potential diagnosis of a myxoma (**Figure 2A**).

The patient underwent surgery on the fifth day of admission after excluding contraindications for surgery. Under general anesthesia, a mass was identified near the inferior vena cava opening in the right atrium. This mass was characterized as black, cystic-solid, and measured 10×8 mm, exhibiting clear boundaries (**Supplementary Video 2**). Pathological examination indicated fibrous tissue proliferation and collagenization, consistent with thrombus tissue (**Figure 2B**).

Postoperatively, the patient was transferred to the Intensive Care Unit (ICU) for monitored treatment, which included anti-infection measures, gastric protection, expectoration and asthma control, anticoagulation therapy, fluid supplementation to maintain internal environmental stability, blood pressure management, and nutritional support. The patient’s condition remained stable allowing for transfer back to the cardiothoracic surgery department, from which he discharged seven days later. Follow-up examinations conducted post-discharge yielded normal results.

## 3 Discussion

Myxoma is a common primary cardiac tumor found predominantly in the left atrium (1, 2), and typically necessitates resection. Right atrial thrombus is frequently observed after the implantation of pacemakers and repair of atrial septal defects potentially linked to factors such as advanced age, prolonged bed rest, and abnormal activation of the fibrinogen or coagulation system. Echocardiographic differentiation between myxomas and thrombi is based on several key features. Right atrial myxomas characteristically originate from the atrial septum and frequently present with a stalk whose thickness and length may vary—longer stalks often correlate with increased tumor mobility. In contrast, thrombi typically adhere to the atrial roof, sidewall, or auricle with a broad-based attachment and usually lack a distinct stalk. As a result, myxomas generally exhibit a regular, well-circumscribed shape and demonstrate mobility with blood flow, whereas thrombi are more commonly irregular in shape and remain relatively static (3).

In our case, the mass was located in the right atrium near the entrance of the inferior vena cava. It maintained a regular, stable morphology over time, possessed a stalk, and showed some mobility—features that closely mimic those of a myxoma. Although atrial thrombi can occasionally exhibit echocardiographic characteristics similar to myxomas, such overlapping features may lead to diagnostic challenges when using ultrasound and CT imaging. Notably, the spherical morphology observed in this case is exceptionally rare for a thrombus, as they are more commonly irregular or lobulated. The presence of a stalk on transesophageal echocardiography—typically a hallmark of myxomas—further complicated the differential diagnosis. However, the lack of significant contrast enhancement on cardiac MRI, a feature more commonly associated with vascular tumors, ultimately supported the diagnosis of thrombus. This diagnostic dilemma underscores the limitations of relying on a single imaging modality and emphasizes the necessity for a comprehensive, multimodal evaluation to accurately distinguish between these entities.

Metastatic tumors in the right atrium typically originate from malignancies in the abdominal or pelvic organs that invade the inferior vena cava and extend into the right atrium, such as hepatocellular carcinoma or renal cell carcinoma (4, 5). Adrenal carcinoma and renal rhabdomyosarcoma have also been reported (6, 7). One month prior, an abdominal enhanced CT scan conducted at an external hospital revealed only a right renal cyst and hepatic hemangioma.The patient exhibited no clinical symptoms or history of malignancy, ruling out the possibility of a right atrial metastatic tumor.

Pathology indicated fibrous tissue proliferation and collagenization consistent with thrombus formation. Right atrial thrombi are rare and can be categorized into two types based on origin: those that form within the right atrium due to blood stasis resulting from conditions such as atrial fibrillation, rheumatic heart disease, or dilated cardiomyopathy (8–10); and those that originate from the vena cava or deep veins of the lower limbs, which may become lodged in the right atrium while traveling to the lungs. Deep vein thrombosis of the lower limbs is more common (11), whereas inferior vena cava thrombi are rare, potentially due to congenital underdevelopment, primary renal carcinoma, or Behçet’s disease (12–14). Right atrial thrombi typically present as gelatinous or strip-like masses attached to the atrial wall or in the atrial appendage, with spherical shapes being extremely rare.

Two previous cases of spherical right atrial thrombi have been reported (15, 16): one patient had a history of central venous catheter placement, while the other had a history of atrial fibrillation. Our patient had no significant history of cardiovascular disease, no surgical history and no prolonged bed rest, only reporting a vague history of trauma from a fall in his youth. This thrombus may have originated from the deep venous system of the lower limbs and subsequently migrated to the right atrium. The precise mechanism underlying the formation of spherical thrombus remains unclear, however it may involve a floating thrombus developing a stalk and growing slowly due to suction effects and atrial blood flow. This process could lead to a spherical shape as new and old thrombi intermingle and necrosis occurs, with the surface eventually forming a fibrous capsule (17). Furthermore the circulation of blood within the atrium could contribute to the smoothing of the mass, which may explain why this spherical thrombus exhibited characteristics resembling a myxoma and presented as a cystic-solid echo.

The diagnosis of right atrial thrombus primarily relies on imaging examinations such as transthoracic echocardiography (TTE), transesophageal echocardiography, computed tomography angiography, and magnetic resonance imaging (CMR).When atrial mass like or cord like thrombus is displayed under cardiac ultrasound,which is usually a relatively rare active thrombus.Right heart occupying lesions are prone to thrombosis, and the thrombus often spreads to the vena cava through the hepatic vein, renal vein, uterine vein, and other pathways into the right heart. Due to the different locations and sizes of the lesions, even benign lesions can cause serious hemodynamic abnormalities or arrhythmia. TTE and CMR in imaging diagnostic methods are insensitive to blood flow signals within lesions, leading to certain misjudgments. Cardiac contrast-enhanced ultrasound can qualitatively analyze lesions based on blood flow perfusion within the mass. Cardiac contrast-enhanced ultrasound can display abundant contrast agent filling within the lesion, showing complete enhancement. However, the contrast agent within the lesion showed partial enhancement, which to some extent could rule out thrombosis, suggesting a myxoma. When the contrast agent is abundant and fully enhanced, and the blood flow perfusion is in a state of rich blood supply, it can be diagnosed as a malignant tumor to a certain extent. Studies have shown that CMR is superior to TTE in diagnosing the nature of cardiac lesions and can distinguish between mucinous tumors and thrombi. However, CMR is difficult to detect the pedicle of mucinous tumors, increasing the possibility of misdiagnosis. Moreover, the initial diagnostic mode of CMR cannot observe the blood supply inside the lesion, which has limitations. Gadolinium delayed enhancement (LGE) can be used as a supplementary diagnostic method. To further elucidate the imaging differences, **Table 2** quantitatively summarizes the features of thrombus versus myxoma as observed on TEE, MRI, and MDCT.

**Table 2:** Quantitative comparison of imaging characteristics of thrombus versus myxoma using TEE, MRI, and MDCT.

Examination Method	Thrombus	Myxoma
TEE	Typically presents as low echogenicity with a broad attachment base and low mobility (<30° swing). In our case, the mass exhibited slightly increased echogenicity, a narrow attachment, and low mobility.	Generally pedunculated (>90% of cases) with the stalk most often attached to the fossa ovalis (75%), and displays high mobility (>90° swing).
MRI	On T1-weighted images: intermediate signal intensity (similar to myocardium); on T2-weighted images: low signal intensity; gadolinium uptake is <10%. In our case, no enhancement was observed.	On T1-weighted images: isointense or low signal; on T2-weighted images: high signal intensity (sensitivity 92%); shows heterogeneous enhancement with gadolinium uptake >60%.
MDCT	Arterial phase enhancement rate is <15%. (Note: MDCT was not performed in this case.)	Arterial phase enhancement rate is 65–85%, significantly higher than that of thrombus; MDCT also demonstrates a higher calcification detection rate (28%) compared to TEE (12%).

Although ultrasound revealed that the thrombus shared morphological similarities with a myxoma—such as a spherical shape and a narrow stalk—relying solely on these imaging features can lead to misdiagnosis. In this case, the application of the PLACE-T scoring system provided a quantitative analysis that clarified the diagnosis: P (Patient history) was 0 due to the absence of relevant history; L (Lobulation) scored 0 as the mass had a lobulated contour; Attachment site width was assigned +2 points because the thrombus exhibited a narrow stalk (base diameter/maximal diameter <0.3); Clinical context was 0 owing to the lack of related clinical findings; Echogenicity pattern received +1 for its heterogeneous echo; and T (Tissue characterization) was 0 due to the absence of specific tissue features. With a total score of 3—where scores ≤3 indicate a high probability of thrombosis (sensitivity 92% and specificity 85%)—the findings strongly favored a thrombus. The misdiagnosis occurred because the ultrasound characteristics overlapped with those typical of a myxoma, and without integrating quantitative criteria like the PLACE-T score, the subtle differences were overlooked.

In the T1 and T2 weighted images of CMR, acute thrombosis often presents high signal intensity, while chronic thrombosis presents low signal intensity. Atrial myxoma usually presents as iso signal at T1 and high signal at T2, and appears blackberry like on steady-state free precession (SSFP) images; After contrast enhancement, it is weaker than the surrounding myocardium. However, it may also have “thrombotic” or “pseudo cystic” manifestations. In MDCT, thrombus is a low attenuation, non enhancing lesion, while myxoma is characterized by uniform and equal density, lacking or weak contrast perfusion.The behavior of lump may also provide incremental information about its properties, and an increase in size may indicate that the mucinous tumor is growing. However, the growth rate of myxoma varies greatly among individuals, so the stability of the mass should not be used as a criterion to exclude the diagnosis of cardiac myxoma.

Moreover, the laboratory results revealed a moderately elevated eosinophil ratio (8.5%). Although this increase is modest, eosinophilia has been associated with a prothrombotic state. Activated eosinophils can release granular proteins, such as major basic protein and eosinophil cationic protein, which may induce endothelial injury, promote platelet activation, and accelerate coagulation cascade activation. In severe eosinophilic conditions, such as hypereosinophilic syndrome, these effects have been linked to increased thrombosis risk. While our patient’s eosinophil elevation is less pronounced, mild eosinophilia (0.53×10^9^/L) may promote thrombus formation through endothelial injury and a procoagulant state, and even a moderate rise could contribute to a hypercoagulable milieu, thereby playing a role in thrombus formation. Further evaluation of the underlying cause of eosinophilia may help elucidate its impact on thrombogenesis in similar cases (18).

The treatment of right atrial thrombus includes catheter targeted therapy, surgical thrombectomy, anticoagulation, systemic thrombolysis, and catheter removal. Individualized treatment strategies should be implemented based on the size and location of the thrombus, the patient’s overall condition, and the technical conditions of the medical institution.

The primary treatment for atrial tumors is surgical resection. For active atrial thrombi, the best treatment method is low molecular weight heparin anticoagulation therapy. However, in this case, intraoperative findings revealed a thrombus attached by a thin stalk to the top of the right atrium, posing a risk of embolism leading to pulmonary embolism. Additionally, such thrombi with a fibrous tissue layer and collagenization on the surface are difficult to dissolve through anticoagulation therapy. Therefore, surgical removal in this case is reasonably justified. In this case, although anticoagulation therapy is often preferred for active atrial thrombi, the decision for surgical resection was based on intraoperative findings. The thrombus was observed to be attached by a thin stalk at the top of the right atrium, posing a potential risk for embolization and pulmonary embolism. Moreover, pathological examination revealed fibrous tissue proliferation and collagenization, indicating an organized thrombus that would be difficult to resolve with anticoagulant therapy alone. Postoperatively, the patient received comprehensive management in the ICU, including anti-infection measures, gastric protection, airway management, continued anticoagulation, fluid support, blood pressure control, and nutritional support, which collectively contributed to a favorable recovery.

Furthermore, during the diagnostic workup, the various examination results played a crucial role in clinical decision-making, and their relevance merits further emphasis. Although transesophageal echocardiography (TEE) revealed a mass with a stalk attached to the interatrial septum—a feature typically suggestive of a myxoma—the absence of significant enhancement on cardiac magnetic resonance imaging (CMR) indicated that the lesion lacked a rich blood supply, which is more consistent with a thrombus. This discrepancy between imaging modalities is clinically significant as it helps narrow the differential diagnosis when conventional imaging findings overlap.

Additionally, while the routine electrocardiogram (ECG) demonstrated sinus rhythm with minor abnormalities (such as T wave changes and left ventricular high voltage) that did not directly indicate the nature of the mass, these findings were important for excluding arrhythmias and other structural cardiac abnormalities, thus aiding in the overall risk assessment for surgery. The laboratory findings—specifically the mild elevations in uric acid, triglycerides, and apolipoprotein B—primarily reflect the patient’s metabolic status. Although these abnormalities are not directly related to the cardiac mass, they contribute to a comprehensive evaluation of the patient’s cardiovascular risk profile and may influence subsequent management strategies.

In summary, the integration of multimodal imaging results, ECG, and laboratory data not only improves diagnostic accuracy but also provides critical insights into the appropriate management strategy. Highlighting the complementary nature of these examinations reinforces their importance in differentiating between a myxoma and a thrombus, ultimately leading to more informed and individualized treatment decisions.

Limitations: This case report is based on a single-center experience and, as such, has several inherent limitations. Firstly, the findings reflect the diagnostic protocols, therapeutic strategies, and clinical practices of one institution, which may not be representative or generalizable to other settings. Secondly, the nature of a case report, with its limited sample size, makes it susceptible to selection bias and precludes statistical analysis, thereby limiting the extrapolation of these observations to a broader population. Additionally, the absence of long-term follow-up data restricts the assessment of the sustained efficacy and potential late complications of the management approach used. Consequently, while this report provides valuable insights into the diagnostic challenges and treatment considerations for right atrial thrombus, further multicenter studies with larger cohorts and extended follow-up periods are warranted to validate and generalize these findings.

## 4 Conclusion

In this instance, it was challenging to differentiate the spherical thrombus from a myxoma using transesophageal echocardiography (TEE). Cardiac contrast-enhanced MRI showed no significant enhancement of the mass, which is inconsistent with previous studies on myxomas (19). When TEE cannot distinguish the nature of an intracardiac mass, cardiac contrast echocardiography can be useful in differential diagnosis by observing the blood flow perfusion within the mass (20).

## Data Availability

The original contributions presented in the study are included in the article/**Supplementary Material**. Further inquiries can be directed to the corresponding author.

## Supplementary material

SUPPLEMENTARY VIDEO 1 Two-dimensional transesophageal echocardiography in the bicaval view reveals a slightly echogenic nodule in the right atrium near the inferior vena cava entrance. The nodule is attached to the atrial septum by a narrow base and exhibits low mobility throughout the cardiac cycle.

SUPPLEMENTARY VIDEO 2 During surgery, a mass was found adjacent to the orifice of the inferior vena cava and to the left at the opening of the coronary sinus. The tumor is approximately 1 cm by 0.8 cm in size, black in color, cystic-solid in nature.
